# Calcium from salmon and cod bone is well absorbed in young healthy men: a double-blinded randomised crossover design

**DOI:** 10.1186/1743-7075-7-61

**Published:** 2010-07-20

**Authors:** Marian K Malde, Susanne Bügel, Mette Kristensen, Ketil Malde, Ingvild E Graff, Jan I Pedersen

**Affiliations:** 1 National Institute of Nutrition and Seafood Research, PO Box 2029 Nordnes, N-5817 Bergen, Norway; 2 Department of Human Nutrition, Faculty of Life Sciences, University of Copenhagen, Rolighedsvej 30, DK-1958 Frederiksberg C. Denmark; 3 Institute of Marine Research, PO Box 1870 Nordnes, N-5817 Bergen, Norway; 4 Department of Nutrition, Institute of Basic Medical Sciences, University of Oslo, PO Box 1046 Blindern, N-0316 Oslo, Norway

## Abstract

**Background:**

Calcium (Ca) - fortified foods are likely to play an important role in helping the consumer achieve an adequate Ca intake, especially for persons with a low intake of dairy products. Fish bones have a high Ca content, and huge quantities of this raw material are available as a by-product from the fish industry. Previously, emphasis has been on producing high quality products from fish by-products by use of bacterial proteases. However, documentation of the nutritional value of the enzymatically rinsed Ca-rich bone fraction remains unexplored. The objective of the present study was to assess the bioavailability of calcium in bones of Atlantic salmon (oily fish) and Atlantic cod (lean fish) in a double-blinded randomised crossover design.

**Methods:**

Ca absorption was measured in 10 healthy young men using ^47^Ca whole body counting after ingestion of a test meal extrinsically labelled with the ^47^Ca isotope. The three test meals contained 800 mg of Ca from three different calcium sources: cod bones, salmon bones and control (CaCO_3_).

**Results:**

Mean Ca absorption (± SEE) from the three different Ca sources were 21.9 ± 1.7%, 22.5 ± 1.7% and 27.4 ± 1.8% for cod bones, salmon bones, and control (CaCO_3_), respectively.

**Conclusion:**

We conclude that bones from Atlantic salmon and Atlantic cod are suitable as natural Ca sources in e.g. functional foods or as supplements.

## Background

In recent years, emphasis has been put in producing high quality nutritional products from fish by-products by use of enzymes [1,2]. The bone fraction, which comprises approximately 10-15% of the total body weight of fish (skin not included) is still regarded as waste. The Norwegian fisheries produce more than 600,000 tons of by-products annually [3], which is more than 20% of the gross weight of fish caught and farmed in Norway. Most of the byproducts are used as low quality raw materials for feed production, and about 180,000 tons are dumped into the sea [3]. Thus, scientific documentation of content and use of various components from marine by-products for human consumption is warranted.

Fish bone has a high calcium (Ca) content, and Ca and phosphorus (P) comprise about 2% (20 g/kg dry weight) of the whole fish. The chemical composition of fish bones varies, and in general, oily fish (e.g. salmon) have higher lipid levels, and lower protein and ash levels compared to lean species (e.g. cod) [4]. Also the bone structure differs between species since a large number of teleosts have acellular bone (bone without enclosed osteocytes). Cellular bones are confined to only a few fish groups, e.g. Salmonidae. There is no consensus for the functional role of acellular bone, and even though a lower mineral level probably is correlated to low stiffness, although this has not been verified experimentally [5]. The crystals in acellular bone are smaller and more easily stained than in cellular bone [6]. This higher surface to volume ratio in acellular fish bone is likely to increase the Ca availability compared to cellular bone. In support of this, a higher Ca availability in acellular eel bone compared to the cellular rat bone was described already in 1974 by [7]. Small fresh water fish are often eaten whole, including bones. Ca from such fish has been shown to have comparable absorption to Ca from skimmed milk both in rats [8] and humans [9]. In another study by Jung et al. [10], Ca retention was increased and loss of bone mineral was decreased by fish bone peptide supplementation in ovariectomised rats. A recent study in growing pigs have shown that Ca from salmon, cod and saithe bones is absorbed as efficiently as CaCO_3 _[11]. The study also indicated that fish species and rinsing method may be of importance both for nutritional content and absorption. Ca-fortified foods are likely to play an important role in helping consumers to meet the calcium requirements needed to reduce the risk of osteoporosis. Thus, there is a need for evaluating Ca absorption from different types of fish bone against other commercially available products. To the authors' knowledge, no study has compared Ca absorption from fish bone by-products with other Ca sources in humans.

The objective of the present study was to evaluate bone by-product from fish as a Ca source in humans. The nutritional composition of fish bones was compared to commercially available calcium supplements, and Ca absorption from enzymatically rinsed fish bones from an oily fish species with cellular bone (Atlantic salmon) and a lean fish species with acellular bone (Atlantic cod), was assessed in young men using ^47^Ca whole body counting. CaCO_3 _was used as control.

## Subjects and Methods

### Study design

**Figure 1:**
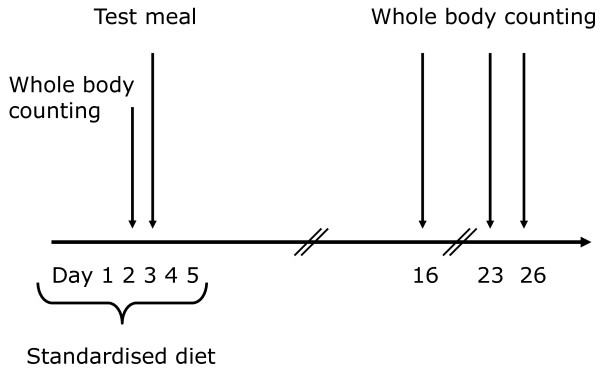
**Time schedule for each of the three test periods starting in November, January and March**. The three test meals contained 800 mg of calcium from three different calcium sources: cod bones, salmon bones and control (CaCO_3_). The calcium absorption was measured in 10 healthy young men using ^47^Ca whole body counting in a double-blinded randomised crossover design.

Three different test meals containing 800 mg Ca from different sources labelled with ^47^Ca were tested in 10 subjects using whole body counting three times during a 4-week period after the ingestion of the test meal. The three test meals were tested according to a double-blinded randomised crossover design, where the subjects were randomly assigned to the sequence of the test meals and there was a wash out period of 7 weeks between the three test meals (Figure 1). To maintain the subjects' calcium intake constant at the time of the test meals, a standardised experimental diet was provided for two days before and three days after ingestion of the test meal corresponding to the individual subjects' habitual Ca intake. In the remaining time between the test meals, the subjects consumed a self-selected diet. The study was approved by the Municipal Ethical Committee of Copenhagen and Frederiksberg (KF-01270319), and the National Institute of Radiation Hygiene, Denmark.

### Subjects

Eleven young healthy men (age: 26 ± 4y; BMI: 24 ± 2 kg/m^2^) were recruited for this experimental study through postings at universities in the Copenhagen area (Table 1). The number of subjects was chosen based on experience from previous studies [12,13]. The subjects were all non-smokers, none of the subjects exercised >10 h/week, took dietary supplements two months prior to start of the study and during the study, were lactose intolerant, had previously participated in studies using radioisotopes, or suffered from any chronic illnesses of relevance to the study. One subject dropped out during the first experimental period for reasons not related to the study. The subjects were given oral and written information about the study, and written consent was obtained from all subjects. After completion of the three meal tests, the subjects received 3000 DKK (US$ ~500).

**Table 1:** Age, body mass index, estimated energy intake, estimated calcium intake (food frequency questionnaire) and chemical analysed calcium content of breakfast given in the experimental period (two days before and two days after ingestion of the test meal), a separate breakfast was provided the test day, containing 800 mg calcium for all subjects.

Subject	Age	Body mass index (kg/m**^2^**)	Energy intake (MJ)	Total Ca intake (mg/d)	Ca content, breakfast (mg/d)	Ca intake, beverages (mg/d)
1	24	20.9	14	1644	1172	22.8
2	22	22.7	14	817	343	24.8
3	22	24.9	15	679	153	44.0
4	27	25.6	18	1512	896	37.0
5	27	28.1	16	1096	582	Not reported
6	34	24.7	16	1909	1362	32.9
7	26	21.6	14	1215	736	29.1
8	26	23.7	12	456	71	Not reported
9	27	24.7	18	1675	1053	43.5
10	22	23.2	12	1456	1038	31.8

### The standardised experimental diet

To keep the subjects' Ca intake constant, a standardised experimental diet was provided two days before the test meal and three days after. The diet was designed to match the subjects' individual habitual calcium intake, which was assessed using a food frequency questionnaire aimed at calcium intake [14] and had an energy distribution corresponding to the habitual Danish diet (15 E% from protein, 35 E% from fat and 50 E% from carbohydrates). The subjects had the same food items for lunch, dinner and snack meal whereas their breakfasts differed due to the different Ca intakes. The diet consisted of common Danish food items such as rye bread, pasta and rice dishes, fruits, juice and candy. Breakfast meals differed and consisted of breakfast cereals, milk, juice, fruit, breads, cheese and jam. The energy intake was estimated by calculation of the subjects basal metabolic rate (BMR = 0.064*W + 2.84) [15] multiplied by a physical activity level (PAL) factor assessed individually based on a questionnaire on physical activity. All meals were prepared in advance in an experimental kitchen unit especially equipped to avoid trace mineral contamination. One meal was daily served at the department and the remaining foods were provided for home consumption where the hot meal could be microwave heated. The subjects were provided with mineral water (14.2 mg Ca/l, Aqua D'or, Brande, Denmark) *ad libitum *which they also were allowed to use for making coffee and tea. The subjects were instructed to register the amount of water consumed daily and were not allowed to drink or eat anything which was not provided for them from the department.

In addition to the standardised experimental diet, the subjects were instructed to take a daily vitamin D_3 _supplement of 10 μg (Naturdrogeriet, Herning, Denmark) for one month before the first test meal and throughout the study period to ensure an adequate vitamin D status. This was necessary as the study ran from October through March were the sun exposure in Denmark is not sufficient to maintain adequate vitamin D production in the skin.

### The test meals, isotopes and labelling procedure

The three test meals contained 800 mg of Ca from three different sources: enzymatically treated cod bones; enzymatically treated salmon bones, and control (CaCO_3_), which were baked into bread rolls. The Ca dose was chosen to reflect a daily dose of a Ca supplement. In addition to the Ca containing bread roll, the test meals consisted of butter and raspberry jam (to camouflage the crunchy fish bones) and was served with 400 ml of ultra pure water. The test persons were monitored during the meal and were given 10-15 minutes to eat. They were instructed to alternate between eating and drinking and to finish their plate. The meal was served at the Department of Human Nutrition, University of Copenhagen on the morning of the test day after a 12 hour fast and the subjects were instructed not to eat or drink for four hours after intake of the test meal.

The isotopes (^47^Ca) were provided by Risø National Laboratory, Radiation Research Department (Roskilde, Denmark) through the Copenhagen University Hospital. The Copenhagen University Hospital received the isotopes the day before dosage of the test meals, prepared doses of 0.2 MBq/ml and transported these to Department of Human Nutrition, University of Copenhagen. Due to problems with the reactor where the isotope was produced, a lower dose was provided in the last period (0.108 MBq ^47^Ca/ml), but this was corrected for in the calculation of calcium absorption. The doses were used to extrinsically label the test meals by drop-wise adding 1 ml of isotope solution (0.2 MBq ^47^Ca) onto the cut surfaces of the rolls. The bread rolls were labelled in the afternoon on the day before intake of the test meal and stored at 5°C until consumption. The external tag method has previously been evaluated [9].

Since each subject received three labelled meals, a maximum dose of 0.6 MBq was received during the study period, which resulted in an estimated maximal radiation dose of 1.5 mSV.

### Whole body counting

The whole-body counter, located at the National University Hospital in Copenhagen, consists of a lead-lined steel chamber with four plastic scintillator blocks (NE110, Nuclear Enterprises Limited, Edinburgh) connected to conventional nuclear electronic modules and a multichannel analyzer system. The counting efficiency and the settings of the energy window were established though measurements of three water-filled phantoms each containing ^47^Ca activity corresponding to a meal and with weights 55, 66, and 77 kg. The individual measurements lasted for 10 min. To minimize contamination by atmospheric background activity, the subjects had a shower and hair wash and were dressed in hospital clothing before each measurement.

### Calcium sources tested

Four different Ca supplements were obtained from a local pharmacy and health store, analysed and evaluated as source for Ca control. The source of Ca in the products differed; being apatite, oyster shell, algae (*Lithothamnion calcareum*), and CaCO_3_. We decided on using CaCO_3 _as control in the present study.

Four different fish bone sources were analysed and evaluated as Ca source for the experimental diets. The sources were salmon and cod bones where soft tissue was removed by use of two different methods, boiling or by use of industrialised produced enzymes. The fish bones rinsed by use of enzymes were chosen as Ca source in the present study. The cod bone was provided by Maritex (Sortland, Norway), and the bones had been rinsed enzymatically by use of Papain (6000 NFPU/mg, Biochem Europe, Mons, Belgium). Bones from fresh salmon (*Salmo salar*) were provided by BioMega A/S, Sotra, Norway. The frames were treated at BioMega A/S with industrially produced enzymes (proteases) [2], similar to protamex (Novozymes A/S, Bagsværd, Denmark). The salmon bones were then rinsed for soft tissue in running cold water using forceps. The cleaned bone samples were freeze dried (Hetosicc, Heto, Denmark) for 74 hours and thereafter ground (Knifetec mill, Foss Tecator). A detailed example of the enzymatic process is described elsewhere [1]. CaCO_3 _produced for human consumption (Merck KGaA, Darmstadt, Germany) served as a control source of Ca.

### Chemical analyses

Representative samples of the Ca supplements and fish bone were analysed in duplicate. The total fat content of the samples was determined gravimetrically after extraction with ethyl acetate [16]. The ash concentration was likewise determined gravimetrically by burning all organic substances in a programmable furnace where the temperature was gradually increased from ambient to 550°C and held at this temperature for 20 hours until constant weight. Total nitrogen was determined using a nitrogen element analyser (LECO, FP-528 N-analyser, Leco Corporation Svenska AB, Sweden). Protein was calculated as N × 6.25.

Prior to the determination of the elements, subsamples were submitted to microwave-assisted wet digestion using 2.0 mL HNO_3 _(ultra pure quality) and 0.5 mL H_2_O_2_, in an Ethos Pro microwave system (Milestone, Holger Teknologi, Oslo, Norway). Flame Atomic Absorption Spectrometry (Perkin-Elmer 3300 AAS, Norwalk, CT) was used for the determination of the elements Na, K, Mg, Ca and Fe ([17,18]). Quantification was made by means of external calibration. Hallow cathode lamps (HCL) were used for magnesium, calcium and iron, whereas sodium and potassium were run in emission mode. The effect of the nitrate concentration present in the sample solutions on the Ca signal were tested [17] and no suppression effect was found. The concentration of P was determined by electrothermal atomic absorption spectrometry on a Zeeman Atomic Absorption Spectrometer (Perkin Elmer 4110 ZL, Norwalk, CT) equipped with na THGA graphite furnace and an AS 72 autosampler. Palladium and magnesium were used as matrix modifier. An Agilent Quadrupole ICP-MS 7500c instrument (Yokogawa Analytical Systems, Inc., Tokyo, Japan) was used as a specific detector for the essential elements: Cu, I, Mn, Se and Zn and the non-essential elements As, Cd, Hg and Pb Six points calibration solutions (5-100 μl/L) were prepared daily by appropriate dilution of 1000 mg/L stock solution of the elements in question (Spectrascan, Teknolab AS, Drøbak, Norway). A diluted solution of a 1000 mg/L (in 10% HCl) certified rhodium stock solution (Spectroscan) was added online and served as an internal standard. Iodine was determined by ICP-MS after the samples were extracted in tetramethylammonium hydroxide (TMAH) for 3 h at 90 °C ([19,20]). The elemental analyses are all accredited by the Norwegian Metrology and Accreditation Service. The certified reference materials TORT-2, Lobster hepatopancreas (National Research council of Canada) and NIST Oyster Tissue (National Institute for Standards and Technology, Gaithersburg, MD, USA) were used for quality assurance of the determination of all elements analysed.

Total Ca content of duplicate portions of the experimental diets and test meals was analysed after homogenisation and microwave digestion (MES-1000, CEM Corporation, Matthews, North Carolina, USA) with HNO3 65%, suprapur (Merck, Darmstadt, Germany) and H2O2 30%, suprapur (Merck, Darmstadt, Germany) by atomic absorption spectroscopy SpectraAA-200 VARIAN (Varian Techtron Pty. Limited, Victoria, Australia) after dilution with lanthaniumoxide solution (Merck, Darmstadt, Germany) (lanthaniumoxide solution: 0.5% lanthaniumoxide, 1% HNO3 and H2O). Standards were prepared from a 1000 mg/L Ca standard (Tritisol^®^, Merck, Darmstadt, Germany) by dilution with lanthaniumoxid solution. A reference diet (Standard Reference Material 1548a, Typical Diet, National Institute of Standards and Technology, Gaithersburg, MD, USA) was analysed in the same run. Analysed Ca content 1.943 ± 0.038 mg/g; certified value: 1.967 ± 0.113 mg/g. The coefficient of variation was 2.0% (n = 4).

Concentration of 25(OH)D was quantified using ^125^I RIA kit (DiaSorin, Stillwater, USA) with an intra-assay of 10% and both cholecalciferol and ergocalciferol metabolites were measured. Intact PTH was quantified with an Immulite intact PTH immunoassay kit (Diagnostic Products Corporation, Los Angeles, Calif.) with an intra-assay precision of 5.8%.

### Calculations and statistics

It has long been known that retention of many bone-seeking elements is inadequately described by a single exponential function, and that a sum of multiple exponentials presents a more accurate picture [21]. Retention (R) after time (t) can then be described by (1):(1)

For large values of t, this function can be accurately approximated by a power function,(2)

where A is the absorption, and b is the rate of excretion.

This approximation reduces the number of parameters that need to be estimated to A and b, and moreover, since the power function is linear in a log-log plot, these parameters can be estimated from empirical data using linear regression on log-transformed values [21,22].

For small values of t, the approximation in (2) is less accurate. It is easy to see that as the exponent b is negative, the retention becomes arbitrarily large as t approaches zero. A more accurate function for small t is also given by Norris et al. [21] as(3)

The parameter y can be estimated empirically; the ICRP's estimate of 0.76 days for Ca (ICRP 1972), is reported to fit well with observed data [23]. However, for estimation it is simpler and more accurate to use the linear approximation (2) and samples taken sufficiently long after ingestion. Beck et al. [22] suggest sampling after day 9. In this study, measurements were taken on day 16, 23 and 26.

Whole body retention of ^47^Ca was measured with a whole-body counter at the Copenhagen University Hospital (Rigshospitalet, Copenhagen). The individual initial radionuclide burden of the subjects was measured before the ingestion of the test meal. Thus, each test person was measured 12 times. All measurements of ^47^Ca were corrected for background radiation and radioactive decay back to the time of administration.

All statistical analyses were performed using the Statistical Analysis System software package, version 9.1 (SAS Institute inc., Cary, N.C.). Dependent variables (absorption % and vit D concentration) were controlled for homogeneity of variance investigation and normal distribution by investigation of residual plots, normal probability plots and histograms. None of the variables required transformation prior to analyses. An ANCOVA was used to examine the effect of treatment on absorption %. This was performed in PROC MIXED, where subject was modeled as a random variable and vitamin D concentration at baseline of each period as covariates. Period and treatment × period interaction was included as fixed variables. The effect of period on vitamin D concentration was evaluated by ANCOVA, where subject was modeled as a random variable and period as a fixed variable. Posthoc pairwise comparisons were made using Tukey-Kramers adjustment. All data are presented as LS means ± SEE unless otherwise stated and the statistical significance level is defined as p < 0.05.

## Results

### Calcium supplements and fish bone meals

The Ca content of the four supplements varied (Table 2). The two products containing apatite and oyster shell were low in Ca, containing 0.04 and 24 g Ca/kg, respectively. The supplements containing algae (336 g Ca/kg) and calcium carbonate (324 g Ca/kg) had a higher Ca concentration. Contrary to the calcium supplements, the fish samples also contained fat and proteins. It should be recorded that the salmon and cod frames treated with enzymes had a higher Ca content and a lower fat content compared to the salmon and cod frames rinsed by boiling. Also, some differences in the concentration of various trace elements between the two differently rinsed bones were found (Table 2). The concentration of the non-essential elements in the salmon and cod frames was low. For the present trial, CaCO_3 _was chosen as control, while cod and salmon bones rinsed by use of enzymes were used as Ca source in the experimental groups.

**Table 2:** Proximal, mineral and elemental composition of different Ca sources.

Component	Salmon bone (protease)^1^	Salmon bone (boiling)^1^	Salmon bone (protease)	Cod bone (boiling)	Calcium supplement from Pharmacy or health store			
					A	B	C	D^1^
Ash (g/100 g)	55.5	43	67.8	65.7	0	2.8	90.9	78.9
Protein (g/100 g)	36	35.7	26.6	32.4	0.46	0.50	0.74	0.56
Fat (g/100 g)	3.0	17.8	<0.2	7.9	0	0	0	0
								
*Minerals (g/kg dry weight)*								
Ca	208	157	261	248	0.04	24	336	324
K	1.3	3.2	0.03	2.3	0.069	0.083	1.3	0.095
Mg	4.1	2.6	3.2	3.5	0.004	0.085	29	1.8
Na	1.9	3.1	2.1	6.0	0.024	0.28	5.9	0.27
P	156	89	151	180	<0.04	<0.04	0.95	0.77
								
*Essential trace elements (mg/kg dry weight)*								
Cu	17.1	4.5	0.5	3.9	<0.3	<0.3	1.0	0.6
Fe	24	20	69	19	< 0.5	7.0	3500	150
I	0.28	<0.02	0.14	3.7	n.d.	0.11	16.2	0.06
Mn	66	36	3.2	28	<0.02	4.2	141.8	15.5
Se	0.3	0.4	0.5	0.4	<0.1	<0.1	0.2	<0.1
Zn	174	106	57	58	<0.1	0.5	8.4	1.9
								
*Non-essential elements (mg/kg dry weight)*								
As	0.34	1.5	0.11	0.61	<0.01	0.01	2.53	1.07
Cd	<0.01	<0.01	<0.01	<0.01	<0.01	<0.01	0.12	0.08
Hg	0.01	0.02	<0.03	<0.01	<0.01	<0.01	<0.01	<0.01
Pb	0.11	0.05	<0.04	0.06	<0.02	0.03	3.03	0.09

Fish bones were rinsed for soft tissue by boiling or use of industrialised produced enzymes (proteases).

^1^Malde et al. [11].

### Calcium absorption using fish bone powders as calcium source

**Figure 2:**
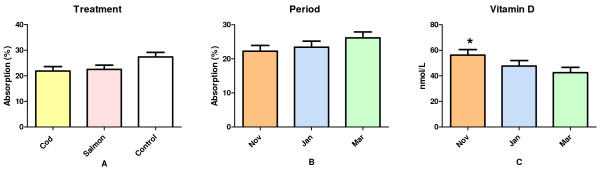
**Results of Ca absorption and vitamin D status in 10 healthy young men**. The calcium absorption was measured using ^47^Ca whole body counting in a double-blinded randomised crossover design. In Figure 2A, Ca absorption from the three different Ca sources; cod bones, salmon bones, and control (CaCO_3_), respectively, is given. In Figure 2B, Ca absorption in each period independent of treatment is given. Vitamin D concentrations in the young men studied, regardless of treatment are given in Figure 2C. All results are mean values ± SEE. * indicate significant differences.

Mean Ca absorption from the three different sources were 21.9 ± 1.7%, 22.5 ± 1.7% and 27.4 ± 1.8% for cod bones, salmon bones, and control (CaCO_3_), respectively (Figure 2A). The individual data is given in Table 3. There was a tendency towards an effect of treatment on Ca absorption after adjusting for vitamin D status and period (p = 0.072) as the difference in Ca absorption from control and cod bones was close to significant (p = 0.089). No effect of period was seen on mean Ca absorption after adjusting for treatment and vitamin D status (p = 0.33), although it increased from period I through III (Figure 2B). Mean absorption was 22.2 ± 1.7% in November, 23.4 ± 1.8% in January and 26.1 ± 1.8% in March.

**Table 3:** Individual data of Ca absorption (%) of ten men given enzymatically rinsed salmon (B) or cod bones (C) in a test meal.

Subject (N = 10)	Order of test meal administration	Control (A)	Salmon (B)	Cod (C)
1	CAB	26	20	15
2	BCA	27	24	20
3	CAB	28	24	18
4	BCA	25	15	23
5	BCA	30	19	21
6	ABC	23	23	30
7	CAB	22	32	26
8	ABC	21	24	23
9	CAB	30	25	22
10	ABC	-	20	18

CaCO_3 _(A) was given as control.

The vitamin D status (25(OH)-D) of the subjects decreased significantly during the study period (P < 0.01) despite a daily supplementation for which reason it is included in the statistical analyses as a covariate. Vitamin D concentrations were 56.3 ± 4.2 nmol/l (November), 47.7 ± 4.3 nmol/l (January) and 42.5 ± 4.2 nmol/l (March), and posthoc analyses showed that vitamin D concentration in November was significantly higher compared to both January (p = 0.025) and March (p < 0.01), whereas the vitamin D status in January and March did not differ (p = 0.20) (Figure 2C).

The levels of parathyroid hormone (PTH) was analysed in the start of the study. The results were within normal range (data not shown).

The average daily energy intake was 14.9 ± 2.1 MJ. The age, body mass index, estimated energy intake, estimated calcium intake and chemical analyses of calcium content of breakfast are given in Table 1.

## Discussion

Ca from the two types of fish bone was absorbed equally well, and the absorption was not significantly different from the control (Figure 2A). The present findings are in accordance with the study by Hansen et al. [9] where Ca absorption from small fresh-water fish was studied. A Ca absorption between 23 and 27% is considered to be within the normal range for calcium salts [24], although several studies also report higher absorption rates [22,23,25]. A meta-analysis of 15 studies (184 healthy subjects) indicated that Ca absorption is highest when Ca source is taken as part of or together with a meal [26]. In the present study, Ca absorption ranged from 15 to 32% (Table 3), with mean values of 21.9 and 22.5% for cod and salmon bones, respectively, and 27.4% for the control (Ca CO_3_).

An interesting observation in the present study is the effect of period on Ca absorption (Figure 2B). This increase cannot be due to carry-over effect of ^47^Ca, as the calculations of absorption compensate for radiation by including a whole body measurement the day before the subjects receive the test meal. Also, background radiation is accounted for in the mathematical model for calculation of absorption. The periodic increase in Ca absorption is also in contrast to a periodic reduction in vitamin D status (Figure 2C) during the study period despite vitamin D supplementation. The subjects were given 10 μg of vitamin D daily from 4 weeks prior to the first test meal and throughout the study period to maintain a serum level of 25OH-vitamin D of 50 nmol/L. The subjects were closely followed by the staff, and all subjects claimed to have taken their daily supplement. A sample of the supplement given was analysed 25 months after study termination to test the vitamin D concentration. The analyses showed that the tablets on average contained 9.1 μg vitamin D. Thus, the observed decreases in vitamin D status was unexpected since supplementation during winter has been associated with increase in serum concentration [27], also for younger men [28]. Our findings are however in line with recent evidence suggesting that the daily vitamin D requirement for maintenance of a 25OH-vitamin D level ≥ 50 nmol/l might be as high as ~30 μg/day [29]. As the three different treatments were given in random order to the subjects, the observed decrease in vitamin D status and the periodic increase in the Ca absorption are not believed to have influenced the absorption differences observed between the different treatments.

The external tag method was not verified in the present study, but we assume that the added ^47^Ca equilibrated with the Ca, naturally occurring in fish bones. In Hansen et al. [9], the external labelling method of small soft-boned fish was evaluated by an *in vitro *method and compared to skimmed milk. It was concluded that the extrinsic tag method was valid for small fish containing bone, although the test also indicated that some of the native Ca did not equilibrate completely with the external ^47^Ca either in the milk or the fish.

Several studies have compared different sources of Ca, and there is no single compound pointed out as a better source than others. However, phosphate containing Ca supplements have been suggested as preferable to carbonate or citrate salt [30] in the treatment of osteoporosis. Fish bones have a naturally high content of both Ca and P, although content varies with species, bone type (acellular vs cellular) and rinsing method. The ash content is highest in lean fish species with acellular bones, e.g. cod and saithe, compared to salmon bones. The protein content is quite similar, while salmon bones contain more fat than cod and saithe bones (Table 2). In the present study, the difference in nutritional composition did not appear to affect on the absorption.

Ca content is an important factor when considering how well a raw material is suitable as food supplements. If the Ca content is too low, a tablet becomes too large to be swallowed, or the number of pills in order to obtain an adequate intake of Ca will be too high. CaCO_3 _is the Ca salt most widely used as a nutritional supplement because of its high elemental calcium content (approximately 40%). In this study we also wanted to compare fish bones with commercially available calcium supplement. Four different Ca supplements were therefore bought in a local health store and pharmacy, and the content of different components were analysed chemically. Two of the Ca supplements contained only traces or small amounts of calcium (Table 2). This was surprising since the supplements were supposed to contain apatite and oyster shell. Most likely these supplements are meant to enhance calcium absorption rather than providing Ca. The two supplements containing either algae (*Lithothamnion calcareum*) or CaCO_3 _had high calcium content, and the algae product had in fact higher Ca concentration than declared (34% Ca compared to 28% Ca). The calcium content of the fish bone samples tested varied from 16 and 26% and was highest in the lean fish species with acellular bone structure rinsed by use of industrialised produced enzymes (Table 2). Thus, fish bones have a slightly lower Ca content than, CaCO_3 _but comparable with other Ca salts like Ca-citrate (Ca_3_(C_6_H_5_O_7_)_2_), Ca-gluconat (C_6_H_11_O_7 _× 1/2 Ca), Ca-lactate (Ca(C_3_H_5_O_3_)_2_) and Ca-acetate (Ca(C_2_H_3_O_2_)_2_) ranging from 9 to 25% [31], and higher than two of the commercially available Ca supplements analysed in the present study.

CaCO_3 _supplement was chosen as control in the present study since it is considered a generally accepted reference calcium source in digestibility studies [32]. The fish bones rinsed by use of industrialised produced enzymes were chosen as calcium source in the experimental groups because they had a higher calcium content than bones rinsed by boiling and because previous studies showed a tendency of better absorption [11].

For an efficient utilisation, fish bones need to be softened. Bones may be softened by heating or treatment with acid [33-35] and the softening increases with cooking time [36]. The softening of fish bone cooked in water can be explained by the elution of a small quantity of the bone proteins into the water, resulting in a change in the bone texture [36]. Softening by cooking to facilitate mastication may contribute to an increased dietary Ca uptake. The fish bones used in the present study were heated during the enzymatic treatment, but not to boiling temperature. In a previous study on pigs [11], enzymatically treated salmon bones resulted in higher Ca absorption than salmon bones boiled for 15 minutes. However, it is possible that even enzymatically treated fish bones may be further softened by heat treatment. Measurement of inorganic components of fish bone after heating revealed that minerals such as Ca, P and Mg were mostly retained in the bone [37]. Thus, heating the bone samples for a longer period may enhance availability of Ca further.

## Conclusion

In conclusion, fish bones are a high value by-product from the fish farm industry and due to the high calcium content this resource can conveniently be utilised as a high quality food ingredient or supplement. In the present work, the calcium in enzymatically rinsed bones from Atlantic salmon and Atlantic cod was demonstrated to be a well absorbed source of Ca in young, healthy men.

## Competing interests

The authors declare that they have no competing interests.

## Authors' contributions

MKM and JIP initiated the study and together with SB and IEG were responsible for the detailed planning of the study. MK and SB were responsible for conducting the trial, and MK also performed the statistical analyses. KM was responsible for applying the Ca retention model. MKM drafted the manuscript, and all authors contributed to the interpretation of the results and final writing and approval of the manuscript.
